# Regulation of Spine Density and Morphology by IQGAP1 Protein Domains

**DOI:** 10.1371/journal.pone.0056574

**Published:** 2013-02-18

**Authors:** Ignacio Jausoro, Ivan Mestres, Gonzalo Quassollo, Lujan Masseroni, Florencia Heredia, Alfredo Caceres

**Affiliations:** 1 Laboratory of Neurobiology, Instituto Mercedes y Martín Ferreyra, INIMEC-CONICET, Córdoba, Argentina; 2 Universidad Nacional de Córdoba, Córdoba, Argentina; University of Toronto, Canada

## Abstract

IQGAP1 is a scaffolding protein that regulates spine number. We now show a differential role for IQGAP1 domains in spine morphogenesis, in which a region of the N-terminus that promotes Arp2/3-mediated actin polymerization and branching stimulates spine head formation while a region that binds to Cdc42 and Rac is required for stalk extension. Conversely, IQGAP1 rescues spine deficiency induced by expression of dominant negative Cdc42 by stimulating formation of stubby spines. Together, our observations place IQGAP1 as a crucial regulator of spine number and shape acting through the N-Wasp Arp2/3 complex, as well as upstream and downstream of Cdc42.

## Introduction

Coordinated interactions among microtubules, microfilaments and members of the family of small Rho-GTPases are crucially involved in the development and maintenance of axons, dendrites and synapses [1]–[4]. IQ motif-containing GTPase-activating protein 1 (IQGAP1) is a 190-kDa scaffolding protein widely expressed among different cell types [5]–[8], including neurons [9]–[11], that links cytoskeletal components with different signaling pathways during cell-cell adhesion, polarization and migration [12]–[16].

However, and despite a well-recognized role for scaffolding and cytoskeletal crosslinker proteins in neuronal polarization and synaptic plasticity [1], [2] the function of IQGAP1 in brain neurons remained largely unexplored until recently. Some of the initial evidences came from a study showing that impairment of N-cadherin-mediated ERK signaling is paralleled by redistribution of IQGAP1 from spines to dendritic shafts [17]. Later on, the same group identified IQGAP1 as a key regulator of dendritic spine number with a specific role in cognitive but not emotional or motivational processes [10]. Mice lacking IQGAP1 exhibited marked memory defects, including impaired long-term potentiation (LTP) in a weak cellular learning model [10]. Interestingly, hippocampal neurons from IQGAP1^−/−^ mice displayed reduced spine number, lower levels of surface NR2A and impaired ERK activity. Other study demonstrated that IQGAP1 and the microtubule plus-end tracking protein, CLIP-170, cooperatively regulate dendritic arbor growth in both cortical and hippocampal pyramidal neurons [11]. Based on these observations and to gain insights into the mechanisms by which IQGAP1 regulates spine morphogenesis, in the present study we used several deletion mutants to identify domains of IQGAP1 that could be necessary for spine and synapse formation.

## Results and Discussion

**Figure 1:**
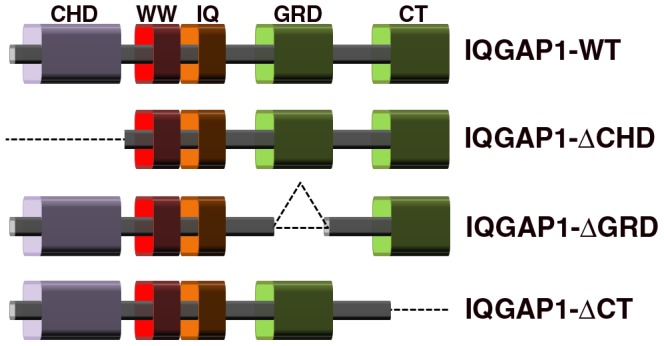
Domain organization of IQGAP1 and representation of the deletion mutants. CHD: Calponin-homology domain. IRS: WW: protein domain containing two highly conserved triptophans that bind proline- rich peptide motifs; responsible for interaction with ERKs. IQ: calmodulin-binding motif; the term refers to the first two amino acids of the motif: isoleucine and glutamine. GRD: Ras GTP related activating protein domain; responsible for interactions with Cdc42 and Rac. CT: C-terminus; responsible for interactions with cadherin, CLIP-170, APC, etc.

IQGAP1 possesses several sequentially arranged functional domains (Figure 1) that enable direct binding to a wide spectrum of cytoskeletal, adhesion and signaling molecules [15], [18], [19]. In this study, we used deletion mutants (Δ) of the following IQGAP1 domains (Figure 1): 1) The Calponin homology domain (Δ-CHD); 2) the RasGAP-related domain (Δ-RGD); and 3) the C-terminal region (Δ-CT), to test their involvement in spine morphogenesis.

**Figure 2:**
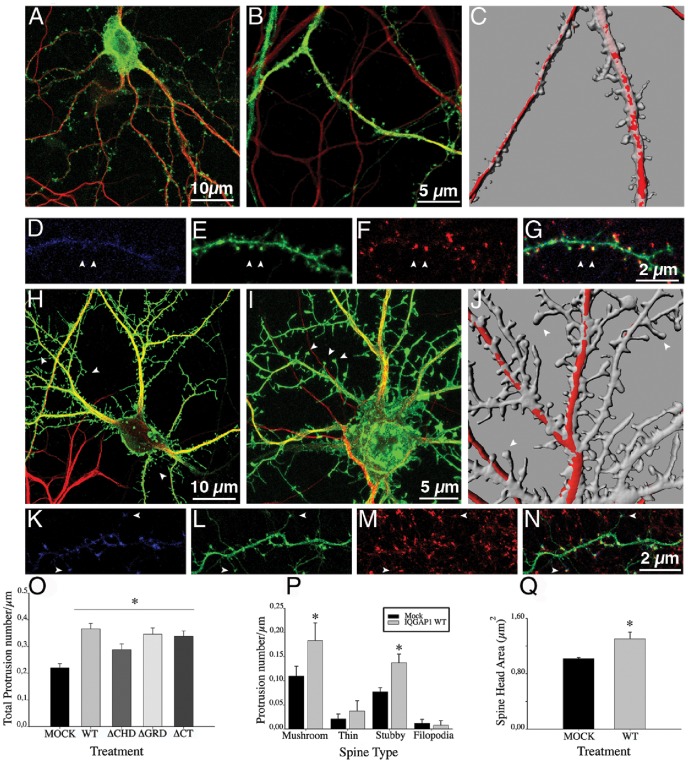
IQGAP1 stimulates spine formation and increases spine head size. (A) Confocal image showing an example of a 17 DIV cultured hippocampal neuron transfected with mock plasmid plus GFP (green) and double stained with MAP2 (red). (B) A high magnification view of a dendritic segment from another neuron of the same culture (C) A 3-D reconstruction of dendritic shafts from a sister culture; note the morphology and density of dendritic spines. (D–G) Confocal images showing a dendritic segment from a neuron co-transfected with myc-tagged-mock plasmid (blue) plus GFP-PSD95 (green) and stained for synaptophysin (red). (G) Merge image; note that GFP-PSD95 (+) spines colocalize with synaptophysin puncta (arrowheads). (H) A Confocal image showing an example of a 17 DIV cultured hippocampal neuron transfected with myc-tagged IQGAP1 WT+GFP (green) and double stained with MAP2 (red). (I) A high magnification view of a dendritic segment from another neuron of the same culture. (J) A 3-D reconstruction of dendritic shafts from a sister culture; note the increase in the number and size of spines. (K–N) Confocal images showing a segment of a dendritic shaft of a neuron co-transfected with myc-tagged-IQGAP1 WT (blue) plus GFP-PSD95 (green) and stained for synaptophysin (red). (N) Merge image. (O–Q) Graphs showing effects of ectopic expression of myc-tagged IQGAP1 WT on spine number, spine type and spine head size. Bars represent mean ± standard deviation. *p<0.0001. The effect of IQGAP1 mutants on spine number is also shown (O).

First, a gain-of-function experiment was performed to evaluate the effect of overexpressing IQGAP1 wild-type (IQGAP1 WT) on spine number and shape. Cultured hippocampal pyramidal neurons were transfected with Green Fluorescent Protein (GFP) or Red Fluorescent Protein (RFP) plus myc-tagged IQGAP1 WT or mock vector 17 days after plating, fixed 18–20 hours later, double stained with MAP2 or synaptophysin and examined by confocal microscopy (Figure 2); in some experiments, neurons were either co-transfected with GFP-PSD95 or stained with a mAb against PSD95. Serial confocal sections were obtained and 3-D reconstructions of dendritic shafts and spines performed using Imaris software. Spine number and type were evaluated by either manual counting or computer-assisted methods using published protocols (see Materials and Methods); both procedures gave similar results (Table S1 and Figure S1). The ectopic expression of myc-tagged IQGAP1 WT (Figure 2) or GFP-tagged IQGAP1 WT (not shown) induced a significant increase in the number of dendritic spines. High-magnification views revealed that neurons overexpressing IQGAP1 WT display many dendritic spines with long necks and large bulbous heads, characteristic of mushroom-shaped spines (Figure 2 H–J). As in the case of mock-transfected neurons these spines localize GFP-PSD95 (Figure 2K–L) or PSD-95 immunofluorescence (Figure S2) to their tips that in most cases was in contact with synaptophysin puncta (Figure 2M–N; Figure S2). Quantitative analysis showed that the increase in spine density of myc-tagged IQGAP1 WT positive neurons was associated with a higher number of mushroom- and stubby-shaped dendritic spines and no significant changes in thin spines and filopodial-like protrusions (Figure 2O, 2P); this analysis also revealed that ectopic expression of IQGAP1 WT induces a significant (p<0.0001) increase in spine head size (Figure 2Q).

**Figure 3:**
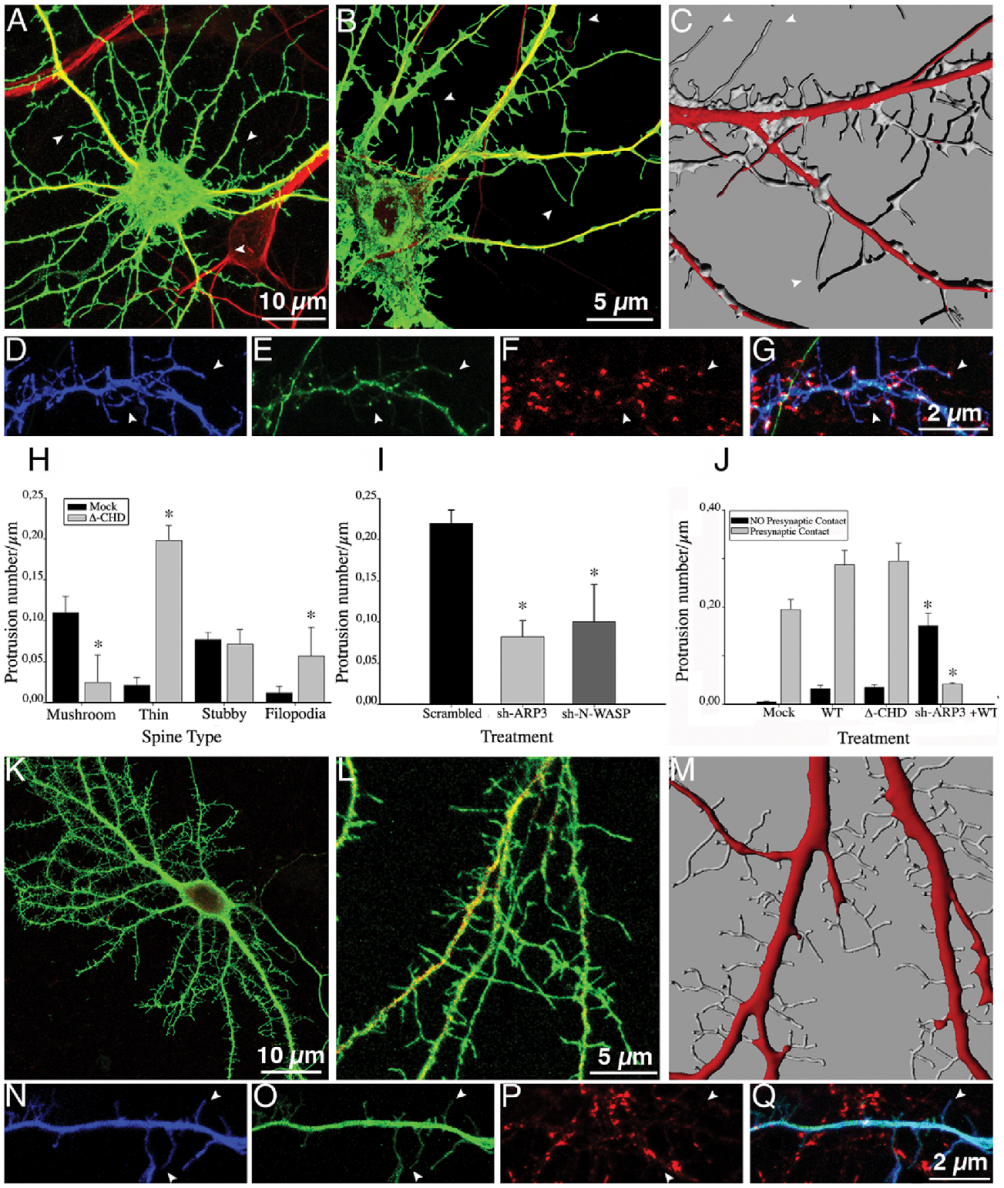
IQGAP1 CHD domain is required for spine head formation. (A) Confocal image showing an example of a 17 DIV cultured hippocampal neuron transfected with myc-tagged Δ-CHD IQGAP1 plus GFP (green) and double stained with MAP2 (red). (B) A high magnification view of dendritic segments from another neuron of the same culture; note the long filopodial-like protrusions merging from dendritic shafts (arrowheads). (C) A 3-D reconstruction of dendritic shafts from a sister culture; note the morphology and density of filopodial-like protrusions (arrowheads). (D–E) Confocal images showing a dendritic segment from a neuron co-transfected with myc-tagged-Δ-CHD-IQGAP1 (blue) plus GFP-PSD95 (green) and stained for synaptophysin (red). (G) Merge image; note that GFP-PSD95 (+) protrusions colocalize with synaptophysin puncta (arrowheads). (H) Graphs showing effects of the ectopic expression of myc-tagged Δ-CHD-IQGAP1 on the number of different types of dendritic spines; note that Δ-CHD-IQGAP1 significantly increases the number of thin spines and filopodial extensions, while decreases the number of mushroom-shaped spines. (I) Graphs showing the effect of scrambled-sh-Arp3, sh-Arp3, and sh-WASP on the number of dendritic protrusions. For this experiment, cultures were transfected with the corresponding GFP-or HcRed-sh plasmids at 17 DIV and fixed 24 h later. Note the significant decrease in the total number of dendritic protrusions in the sh-Arp3 and sh-WASP-treated groups. (J) Graphs showing the number of dendritic protrusions contacting synaptophysin puncta in neurons transfected with IQGAP1 WT, Δ-CHD-IQGAP1 and sh-Arp3 plus myc-tagged-IQGAP1 WT. Note the dramatic decrease in the number of dendritic protrusions contacting synaptophysin puncta in the cells treated with sh-Arp3 plus IQGAP1 WT; most of these protrusion resemble filopodial extensions. (K) Confocal image showing an example of a 17 DIV cultured hippocampal neuron transfected with HcRed-sh-Arp3 (red) plus myc-tagged IQGAP1 WT (green). (L) A high magnification view of a dendritic segment from another neuron of the same culture. (M) A 3-D reconstruction of dendritic shafts from a sister culture; note the presence of many filopodial-like protrusions. (N-P) Confocal images showing a segment of a dendritic shaft of a neuron co-transfected with myc-tagged-IQGAP1 WT (blue) plus GFP-sh-Arp3 (green) and stained for synaptophysin (red). (Q) Merge image. Note that many filopodial protrusions are not contacted by synaptophysin puncta. Bars represent mean ± standard deviation. * p<0.0001.

We then examined the consequences of expressing an IQGAP1 mutant lacking the CHD (Figure 1, Δ-CHD-IQGAP1). The CHD (amino acids 44–159) located at the N-terminus is responsible for actin and presumably N-WASP binding, as well as capable of promoting Arp2/3-mediated actin polymerization and branching [16]. Neurons expressing this mutant display an increase in the number of spine-like protrusions (Figure 2O; Figure 3A). High-magnification views (Figure 3B) and 3-D reconstructions (Figure 3C) revealed that many of these protrusions resembled filopodial-like extensions lacking a discernable head or displaying a small expansion at the tip. Therefore, we tested, whether or not these structures may represent thin spines or conventional filopodia [20]–[24] by co-expressing Δ-CHD-IQGAP1 with GFP-PSD95 or staining with anti-PSD95. Interestingly, co-expression of these constructs revealed that the majority (>95%) of the filopodial extensions display GFP-PSD95 fluorescence at their tips (Figure 3D, 3E) and colocalize with synaptophysin puncta, typical of thin spines (Figure 3F, 3G); quantitative analysis confirmed these observations (Figure 3H–J). Collectively, these results suggest that the CHD of IQGAP1 is required for proper spine head formation, and that in its absence thin spines capable of contacting synaptic terminals are formed; they also raise the possibility that the increase in the number and size of mushroom-shaped spines elicited by IQGAP1 WT could require N-WASP and/or Arp 2/3 expression. Previous work has suggested that the Arp 2/3 complex induces formation of the branched actin network in the spine head [21], [23], [25], [26]. Therefore, we generated sh-RNAs specific for Arp3, Arp2 [27] and N-WASP (Figure S3). As shown in Figure 3I, silencing of Arp3 significantly reduces spine number. The remaining dendritic protrusions resemble filopodial extensions; a similar phenotype was observed after Arp2 (not shown) or N-WASP suppression (Figure 3I). Interestingly, co-expression of IQGAP1 WT in Arp 2, or 3 or N-WASP-suppressed neurons stimulated the formation of filopodial-like extensions, but not of mushroom- or stubby spines (Figure 3K–M). This phenotype resembles the one observed after expression of Δ-CHD; however, the filopodial protrusions formed in the absence of Arp 2/3 or N-WASP failed to localize with synaptophysin puncta (Figure 3J, 3N–Q) and do not contain PSD95 at their tips (not shown). This is consistent with the idea that modeling of the actin cytoskeleton has also a major role in organizing the post-synaptic density [22], [26], [28]. It has also been reported filopodial formation in the absence of Arp2/3 or WAVE [27], [29]. Therefore, the protrusions observed in Arp2/3 or WASP-suppressed neurons co-expressing IQGAP1 WT may represent either immature thin spines or “conventional filopodia”. Since our results also suggest that the CHD is dispensable for stalk/neck formation, we decided to examine the role of GRD.

**Figure 4:**
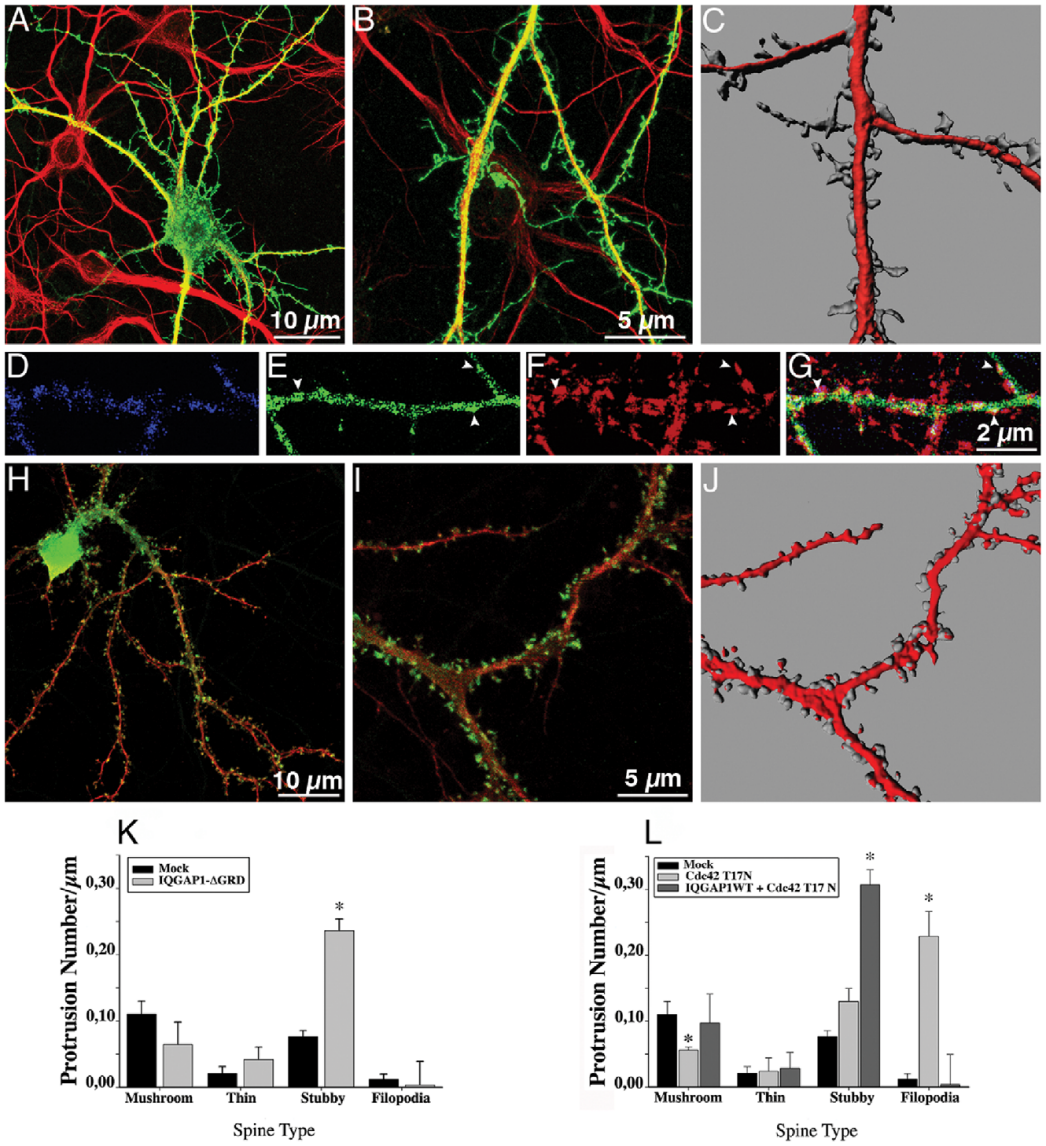
The IQGAP1 GRD domain is required for stalk spine extension. (A) Confocal image showing an example of a 17 DIV cultured hippocampal neuron transfected with myc-tagged Δ-GRD-IQGAP1 plus GFP (green) and double stained with MAP2 (red). (B) A high magnification view of dendritic segments from another neuron of the same culture; note that many of the protrusions lack discernable stalks and are apposed to dendritic shafts. (C) A 3-D reconstruction of dendritic shafts from a sister culture; note the morphology and density of dendritic protrusions that resemble stubby spines lacking stalks or displaying very short ones. (D–F) Confocal images showing a dendritic segment from a neuron co-transfected with myc-tagged-Δ-GRD-IQGAP1 (blue) plus GFP-PSD95 (green) and stained for synaptophysin (red). (G) Merge image; note that patches of GFP-PSD95 colocalize with synaptophysin puncta (arrowheads). (H) Confocal image showing an example of a 17 DIV cultured hippocampal neuron transfected with myc-tagged IQGAP1 WT plus GFP (green) plus HA-tagged DN-Cdc42 (T17N; red). (I) A high magnification view of dendritic segments from another neuron of the same culture. (J) A 3-D reconstruction of dendritic shafts from a sister culture. Note that IQGAP1 WT failed to stimulate the formation of mushroom-shaped spines; most of them resemble stubby spines. (K) Graphs showing effects of the ectopic expression of myc-tagged Δ-GRD-IQGAP1 on the number of different types of dendritic spines; Δ-GRD-IQGAP1 significantly increases the number of stubby spines. (L) Graphs showing effects of the ectopic expression of myc-tagged IQGAP1 WT+HA-tagged T17N on the number of different types of dendritic spines; note the increase in the number of stubby spines. Bars represent mean ± standard deviation. * p<0.0001.

This domain (Figure 1), which extends from amino acids 1025 to 1238 binds to the small GTPases Cdc42 and Rac1, both implicated in spine formation [3], [4]. Expression of IQGAP1 Δ-GRD also increases spine number (Figure 2O and Figure 4A–G, 4K); analysis of spine shape reveals a selective increase in the number of stubby spines (Figure 4K) that contain GFP-PSD95 (Figure 4E) or stain for PSD95 (Figure S2) and are contacted by synaptophysin puncta (Figure 4F–G; Figure S2). These observations suggest that IQGAP1 interaction with Cdc42 and/or Rac could be important for stalk-neck formation; since a recent study has shown that stimulated spines have increased Cdc42 activation at the spine neck [30] we tested the effect of a Cdc42 dominant negative (DN) mutant (T17N) on the stimulatory effect of IQGAP1 WT on spine formation. In agreement with previous observations [26] expression of T17N reduced the number of mushroom-shaped spines, consistent with its proposed role in stalk-neck formation; intriguingly, T17N also induced a significant increase in the number of filopodial extensions (Figure 4L). However, co-expression of IQGAP1 WT reverts the T17N phenotype, stimulating formation of stubby spines and reducing filopodial number (Figure 4H–L), suggesting that IQGAP1-mediated stalk-neck formation and/or extension requires Cdc42. Conversely, spine deficiency caused by IQGAP1 suppression (Figures S4 and S5) can be rescued by co-expression of an active fast cycling mutant (F28L) of Cdc42 [31], [32] (Figure S5). Together, these observations favor the idea that IQGAP1 acts both upstream (e.g. a regulator) and downstream (e.g. an effector) of Cdc42 [18], [33]; they also suggest that Cdc42 may not require IQGAP1 to promote spine formation.

**Figure 5:**
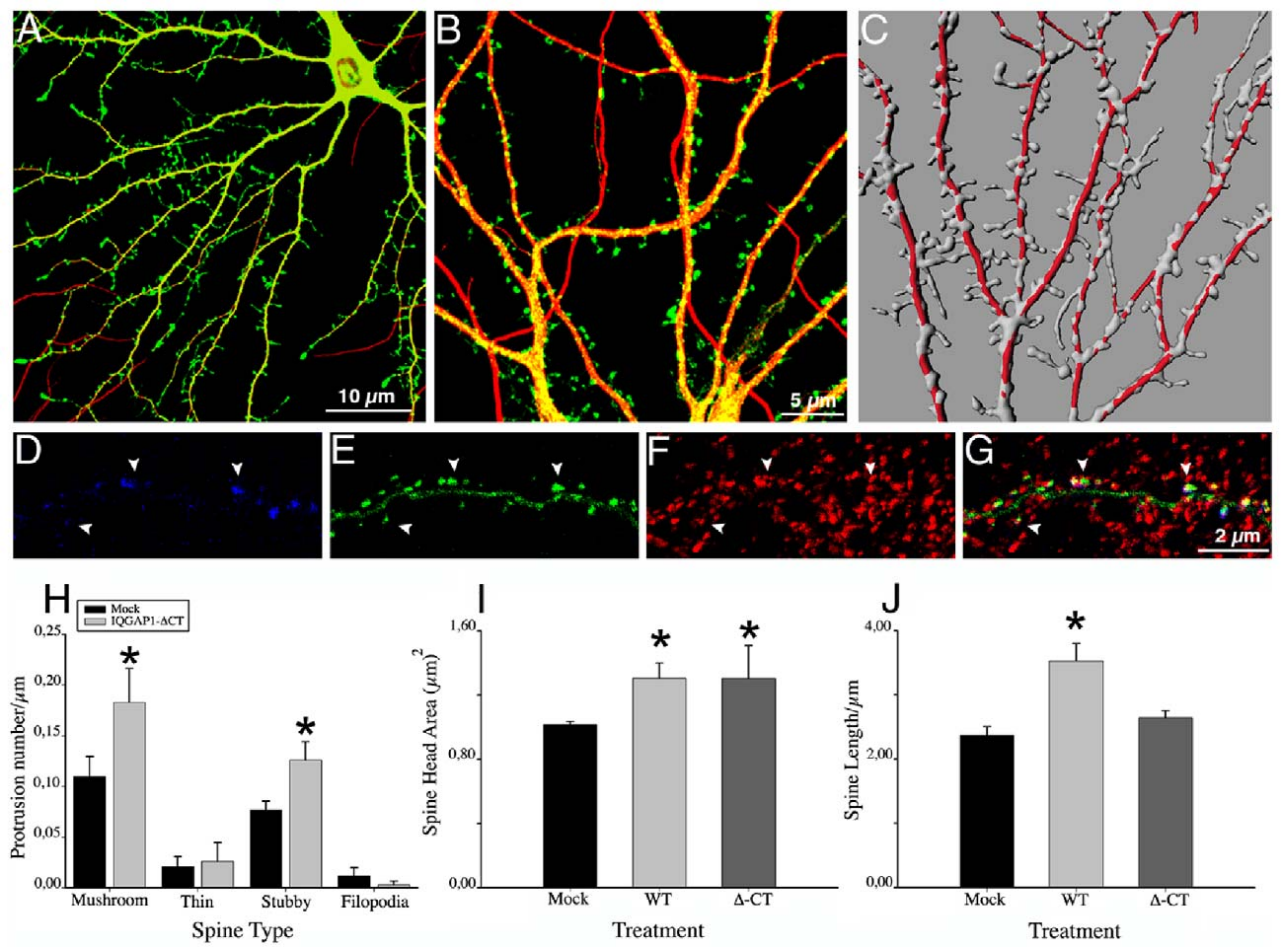
Δ-CT IQGAP1 stimulates spine formation. (A) Confocal image showing an example of a 17 DIV cultured hippocampal neuron transfected with myc-tagged Δ-CT-IQGAP1 plus GFP (green) and double stained with MAP2 (red). (B) A high magnification view of dendritic segments from another neuron of the same culture; (C) A 3-D reconstruction of dendritic shafts from a sister culture. (D–F) Confocal images showing a dendritic segment from a neuron co-transfected with myc-tagged-Δ-CT-IQGAP1 (blue) plus GFP-PSD95 (green) and stained for synaptophysin (red). (G) Merge image. (H–J) Graphs showing the effect of expressing myc-tagged-Δ-CT-IQGAP1 on spine number/type and spine head size and length. Bars represent mean ± standard deviation. *p<0.0001.

Next, we evaluated the IQGAP1 C-terminus (Figure 1, CT). This region, comprising amino acids 1563–1657, binds to several proteins including CLIP-170, adenomatous polyposis coli (APC), and E-cadherin [18]. Since an interaction between IQGAP1 and CLIP170 regulates dendritic growth [11] and dynamic microtubules containing +TIPs invade spines [34], it became of interest to explore if expression of a deletion mutant of IQGAP1 lacking the CT could affect spine number or shape. The results obtained show that deletion of this domain does not prevent the stimulatory effect of IQGAP1 on spine number or spine head size; however, this mutant fails to increase the total length of mushroom-shaped spines, as does full length IQGAP1 (Figure 5).

**Figure 6:**
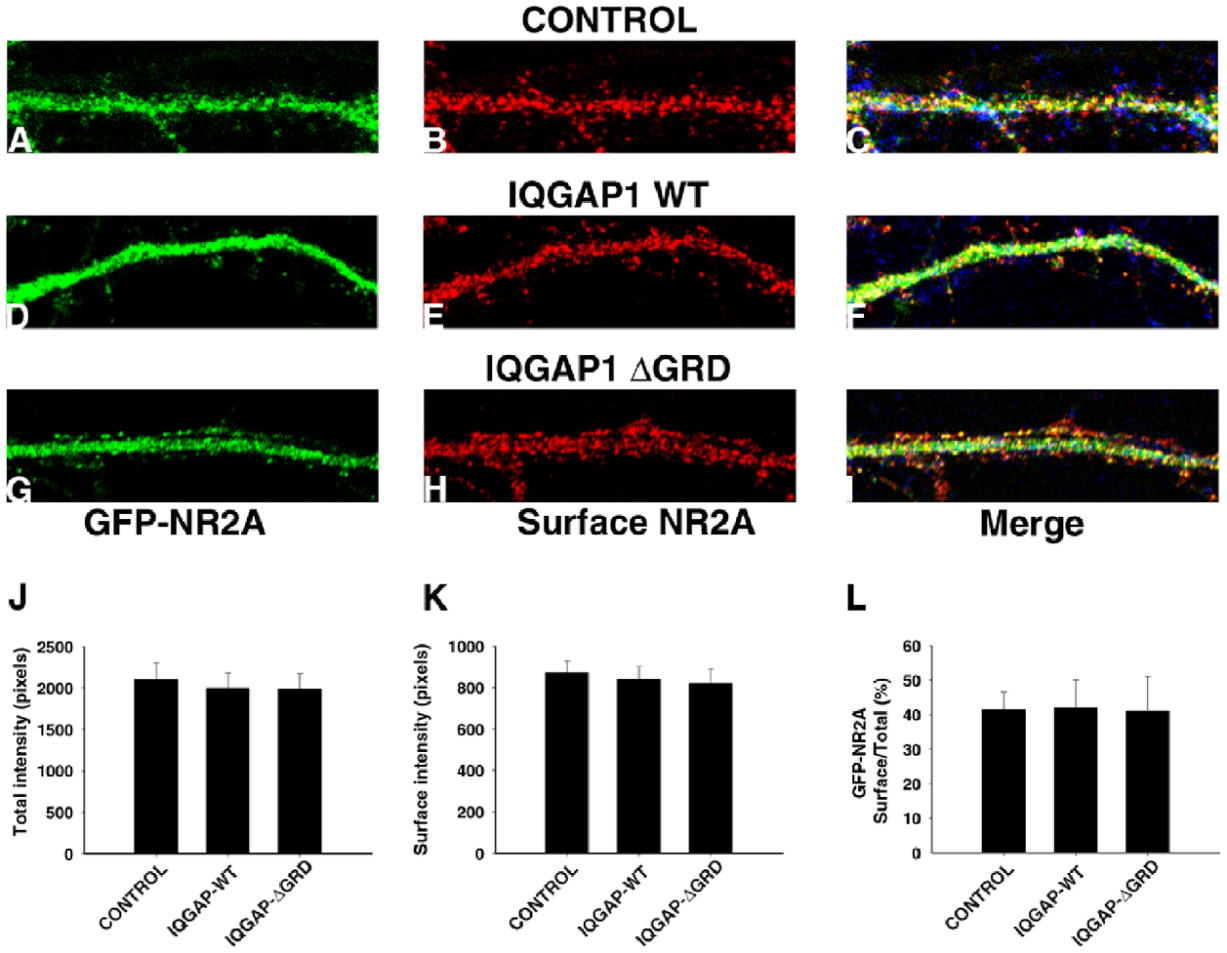
Distribution of NR2A in hippocampal neurons expressing IQGAP1 WT or deletion mutants. (A–C) High magnification views of a dendritic segment from a 17 DIV cultured hippocampal neuron transfected with myc-tagged-mock plasmid (blue in merge) plus GFP-NR2A (green) and double stained with anti-GFP for surface NR2A (red). (D–F) A similar set of images but from a culture transfected with myc-tagged IQGAP1 WT (blue in merge) plus GFP-NR2A (green) and double stained with anti-GFP for surface NR2A (red). (G–I) A similar set of images but from a culture transfected with myc-tagged Δ-GRD IQGAP1 (blue in merge) plus GFP-NR2A (green) and double stained with anti-GFP for surface NR2A (red). Note that in all cases dendritic spines contain NR2A labeling at their tips. (J–L) Quantification of GFP-NR2A total fluorescemce intensity (J), GFP-NR2A surface fluorescence intensity (K), and the ratio of surface vs. total GFP-NR2A fluorescence intensities (L) in neurons expressing myc-tagged mock plasmid (control), myc-tagged IQGAP1 WT and myc-tagged Δ-GRD IQGAP1. No significnt differences were detected between control neurons and those expressing IQGAP1 WT or Δ-GRD IQGAP1. Similar results were observed after ectopic expresson of Δ-GRD IQGAP1or Δ-CT IQGAP1 (not shown). Intensity values (8-bit images) are expressed in pixels. Black = 0/White = 256. For further details see Ref. [35]–[37]. Values represent the mean ± S.E.M. A least 20 dendritic segments (50 µm in length) per cell were measured for each experimental condition.

In the final set of experiments we evaluated the possible participation of the CHD or GRD or CT domains of IQGAP1 in regulating the distribution of the NR2A, a subunit of the NMDA receptor. Previous studies have shown that IQGAP1 interacts with NR2A and that NR2A surface levels were significantly decreased in IQGAP1−/− mice [10]. To test if any of the IQGAP1 deletion mutants used in this study could affect the intracellular or surface distribution of NR2A, we co-transfected GFP-tagged NR2A at the N-terminus with each of the deletion mutants. By staining non-permeabilized transfected neurons with a mAb against GFP, we were able to label surface NR2A [10]. The results obtained (Figure 6) showed that none of the IQGAP1 deletion mutants alter the intracellular or surface distribution of NR2A, suggesting that other IQGAP1 domains or a combination of domains regulate the distribution of this NMDA subunit.

### Conclusions

The present results confirm and extend recent studies [10], [11], [17] indicating that IQGAP1 is required for spine formation. The new information presented here suggests that IQGAP1 protein domains actively participate in spine morphogenesis and differentially affect spine number and shape acting through the N-WASP- Arp2/3 complex and Cdc-42 signaling.

## Materials and Methods

### Short-Hairpins RNA and Plasmid Constructs

The IQGAP1, Arp2, Arp3, N-WASP short hairpin (sh) RNAs and their corresponding scrambled control sequences were constructed using previously described procedures [35], [36]. In brief, DNA fragments containing U6-sh-RNA and U6-scrambled-sh were inserted into pCAG vector in which the GFP or HcRed cDNA is under the control of a chick actin-minimal (CAG) promoter [35], [36].

The following targeting sequences were used:

For IQGAP1-sh

Forward: 5′TGCCATGGATGAGATTGGAAAGCTTTCCAATCTCATCCATGGCACTTTTTTG and.

Reverse: 5′AATTCAAAAAGTGCCATGGATGAGATTGGAAAGCTTTCCAATCTCATCCATGGCA.

For Arp 2-sh

Forward:

5′GGTTGGTGTTGCTGAATTGCTTAAGCTTAAGCAATTCAGCAACACCAACCCTTTTTG.

Reverse:

5′AGCTTAAGCAATTCAGCAACACCAACCAATTCAAAAAGGGTTGGTGTTGCTGAATTGCTTA.

For Arp3-sh

Forward:

5′GGTACAGTAATAGACAGTGGAGAAGCTTCTCCACTGTCTATTACTGTACCCTTTTTG.

Reverse.

5′AGCTTCTCCACTGTCTATTACTGTACCAATTCAAAAAGGGTACAGTAATAGACAGTGGAGA.

For N-WASP-sh

Forward:

5′GGATGCACTTCTAGACCAGATAAAGCTTTATCTGGTCTAGAAGTGCATCCCTTTTTG.

Reverse:

5′AGCTTTATCTGGTCTAGAAGTGCATCCAATTCAAAAAGGGATGCACTTCTAGACCAGATAA.

The resulting plasmids were referred to as sh-IQGAP1-GFP or HcRed, sh-ARP2-HcRed, sh-ARP3-HcRed, sh-NWASP-HcRed, and the corresponding scrambled sh-RNAs. For myc tagged-IQGAP1 WT, RNA was isolated from HEK293 cells, and IQGAP1 transcripts amplified by RT-PCR with the following primers:

Forward (IQ1F): 5′-ATGTCCGCCGCAGACGAGG-3′ and Reverse (IQ1Rev): 5′-TTACTTCCCGTAGAAC-3′.

The DNA was then cloned in pcDNA3.1 Myc. The IQGAP1-Δ-CHD mutant was generated from pcDNA3.1-IQGAP-WT by PCR and sub cloned in pcDNA3.1 by generating a SalI site using the following primers:

Forward: 5′-GTCGACGAGAAGTATGGCATCC and Reverse 5′- TTACTTCCCGTAGAAC-3′.

For IQGAP1-Δ-GRD a silent mutation was generated to create an Nhe1 site to eliminate the GRD region and allow re-ligation. The following primers were used: Forward: 5′-GGAAATCAAGTCGAAGCTAGCTCAGATTCAAGAGATTGTGACAGG-3′ and Reverse: 5′-CCTGTCACAATCTCTTGAATCTGAGCTAGCTTCGACTTGATTTCC-3′. For PSD95-GFP, RNA was isolated from 36 days old rat brains. A transcript was then amplified using the following primers:

Forward: 5′-GGCCCGAATTCATGGACTGTCTCTGTATAG-3′ and Reverse: 5′-GCCAGGGTACCAATCAGAGTCTCTCTC -3′.

The transcript was then cloned into pEGFP C2 between EcoR1 and KpNI.

For IQGAP1-ΔCT a mutation was generated to create a stop codon at position 1377. The following primers were used:

Forward: 5′-GCCTGGAGATGAGAATGCATAAATGGATGCTCGAACC-3′.

Reverse: 5′-GGTTCGAGCATCCATTTATGCATTCTCATCTCCAGGC-3′.

The Cdc42 fast cycling mutant [31] (Clone Cdc42-F28L) and the Dominant Negative (DN, Clone T17N) one were a generous gift from Dr. R. A. Cerione, (Cornell University, Ithaca, New York). The GFP-NR2A cDNA [32] was a generous gift of Dr. B. Vissel (Neurodegenerative Disorders Laboratory, Neuroscience Department, Garvan Institute of Medical Research, Sydney, NSW, Australia).

### Animals, Culture, Transfection, Immunofluorescence, Confocal Microscopy, Morphometry and 3D Reconstruction

Pregnant rats (Wistar, Charles River 251 Ballardvalle Street, Wilmington, MA01887) were obtained from the institutional (INIMEC-CONICET) specific pathogen free (SPF) vivarium. All animal procedures and care were approved by the institutional animal care committee (INIMEC-National Research Council and Universidad Nacional de Córdoba, Argentina) and the National Department of Animal Care and Health (SENASA-Argentina). Efforts were made to minimize animal suffering and to reduce the number of animals used.

Embryonic day-18 rat embryos (euthanized by CO2 overdose) were used to prepare primary hippocampal cultures as previously described [35]–[40]. Neurons of 17 days *in vitro* (DIV) with long axons and well-developed dendritic arbors were used for all experiments [39]. Transient transfection of cultured neurons was performed as described previously [32], [35]–[37], [39], [40] and the constructs used at concentrations ranging from 2 to 4 mg/ml. Neurons were fixed with 4% paraformaldehyde in 4% sucrose-containing PBS and permeabilized in 0.2% Triton X-100 in PBS for 5 min before antibody incubation as described [35]–[40]. Neurons were fixed 18–20 after transfection in the case of cultures transfected with cDNAs or after 24 hours in the case of cells expressing short hairpin RNAi.

The following primary antibodies were used in this study: a monoclonal antibody (mAb) against MAP2 (clone AP-20) diluted 1∶1000; an affinity purified rabbit polyclonal antibody against IQGAP1 (Santa Cruz Biotechnology, H-109, sc-10792) diluted 1∶100 for immunofluorescence or 1∶2000 for immunoblotting; a rabbit polyclonal antibody or a mouse mAb against myc (Santa Cruz Biotechnology) diluted 1∶300; a mAb against synaptophysin (Chemicon International); a mAb against WASP (Santa Cruz Biotechnology) diluted 1∶2000; a rabbit polyclonal antibody against GFP (Molecular Probes, A 1122) diluted 1∶500; a mAb against PSD95 (AbCam, AB 2723) diluted 1∶250; and a rabbit polyclonal antibody against Arp2 (Santa Cruz Biotechnology) diluted 1∶2000. Cells were visualized using a conventional inverted confocal microscope (Zeiss Pascal) or a spectral one (Olympus FV1000). Images were processed using Adobe Photoshop.

Neuronal shape parameters were evaluated as described previously [38]–[40]. Briefly, maximal projection images showing the complete neuronal arbor of transfected neurons visualized by GFP or HcRed fluorescence or myc-tagged IQGAP1 immunofluorescence were created from confocal images acquired through a 60× or 63×, 1.4 NA oil objective. Spine number and shape were assessed manually by GFP or RFP fluorescence or myc-IQGAP1 immunofluorescence and the presence of synapses by co-expressing GFP-PSD95 and/or co-staining with synaptophysin as described by Tolias et al., [41]. We also used computer-assisted methods for evaluating spine number and shape using the procedures described by Rodriguez et al., [42], [43]. No differences in spine number or shape were found between manual and computer-assisted methods; both procedures gave similar results (Figure S1, Table S1). At least 10 dendritic segments (50 µm length/each) per cell (total 6 cells per culture) from at least 3 different cultures were analyzed for each experimental condition. Differences among experimental groups were analyzed by one-way ANOVA and Tukey’s *post hoc* test.

### Western Blot

Changes in the levels of IQGAP1, N-WASP and ARP2/3 after RNA interference treatments of CHO cells or cultured hippocampal pyramidal neurons were analyzed by Western blotting as described previously [35]. Densitometry of Western blots were performed using Scion Image software.

## Supporting Information

Figure S1 **Computer-assisted analysis of spine number and shape.** A representative image of the computer display provided by the software used to automatically evaluate changes in spine number and shape. For further details see Ref. 42 and 43.
(TIFF)

Figure S2 **PSD95 immunofluorescence in neurons expressing IQGAP1 and its mutants.** (A-C) High magnification views of a dendritic segment from a 17 DIV hippocampal cell culture transfected with myc-tagged IQGAP1 WT (green) and double stained with anti-PSD95 (red); note that long spines stain for PSD95 (arrows). (D-F). A similar set of images but from a culture transfected with myc-tagged IQGAP1 WT (green) and double stained with anti-synaptophysin (red); note that spines colocalize with endogenous synaptophysin puncta (arrows). (G-I) High magnification views of a dendritic segment from a 17 DIV cultured hippocampal neuron transfected with myc-tagged Δ-GRD IQGAP1 (green) and double stained with anti-PSD95 (red); note that stubby spines stain for PSD95 (arrows). (J-L) A similar set of images but from a 17 DIV cultured hippocampal neuron transfected with myc-tagged Δ-GRD IQGAP1 (green) and double stained with anti-synaptophysin (red); note that stubby spines colocalize with endogenous synaptophysin puncta (arrows).
(TIFF)

Figure S3 **Short-hairpin RNAi suppression of Arp3, Arp2 and NWASP.** Western blots showing levels of Arp3, Arp2, NWASP, and ß-tubulin in extracts of CHO cells treated with sh-Arp3, sh-Arp2, sh-NWASP or their corresponding scrambled oligonucleotides. For this experiment, cultures were transfected with the corresponding constructs and cell extracts obtained 24 hours later.
(TIFF)

Figure S4 **Short-hairpin RNAi suppression of IQGAP1.** (A) Schematic representation of the sh-IQGAP1. (B) Western blots showing levels of IQGAP1 in extracts of CHO cells treated with sh-IQGAP1 or its corresponding scrambled oligonucleotide (ssh-IQGAP1). (C, D) Confocal images showing representatives images of cells expressing sh-IQGAP1-GFP (green) double stained with anti-IQGAP1. Note the decrease in IQGAP1 fluorescence in the cells expressing the RNAi (arrows). (E) Quantitative measurements of IQGAP1 fluorescence intensity in cultured hippocampal pyramidal neurons transfected with ssh-IQGAP1 or sh-IQGAP1. The values expressed in pixels represent the average fluorescent intensity within the cell body, initial, middle and distal neuritic segments. Note the significant reduction (P<0.001) in IQGAP1 fluorescent intensity in the RNAi-treated cultures. In all these experiments, fluorescent measurements were performed using 8-bites images.
(TIFF)

Figure S5 **Cdc42 rescues the inhibitory effect of IQGAP1 suppression on spine number.** (A) A dendritic segment of a neuron expressing ssh-IQGAP1-GFP (scrambled) oligonucleotide. (B) A dendritic segment of a neuron expressing sh-IQGAP1-GFP oligonucleotide; note the decrease in spine number. (C-F) Graphs showing the effect of IQGAP1 suppression on the number and type of dendritic protrusions. Note that a fast cycling Cdc42 (F28L) mutant rescues the decrease in spine number observed after IQGAP1 suppression. Bars represent mean ± standard deviation.
(TIFF)

Table S1 **A comparison of spine number and type as determined by manual counting vs. automated methods** [**42**], [**43**] **.** Each value is the mean ± standard deviation. At least 10 dendritic segments (50 µm length/each) per cell (total 6 cells per culture) from at least 3 different cultures were evaluated. Note that both methods gave similar results.
(DOC)
