# Supplementary figures and images for: Regulation of Spine Density and Morphology by IQGAP1 Protein Domains
Source: PLoS One. 2013 Feb 18;8(2):e56574. doi: 10.1371/journal.pone.0056574 (PMC3575492; doi:10.1371/journal.pone.0056574)

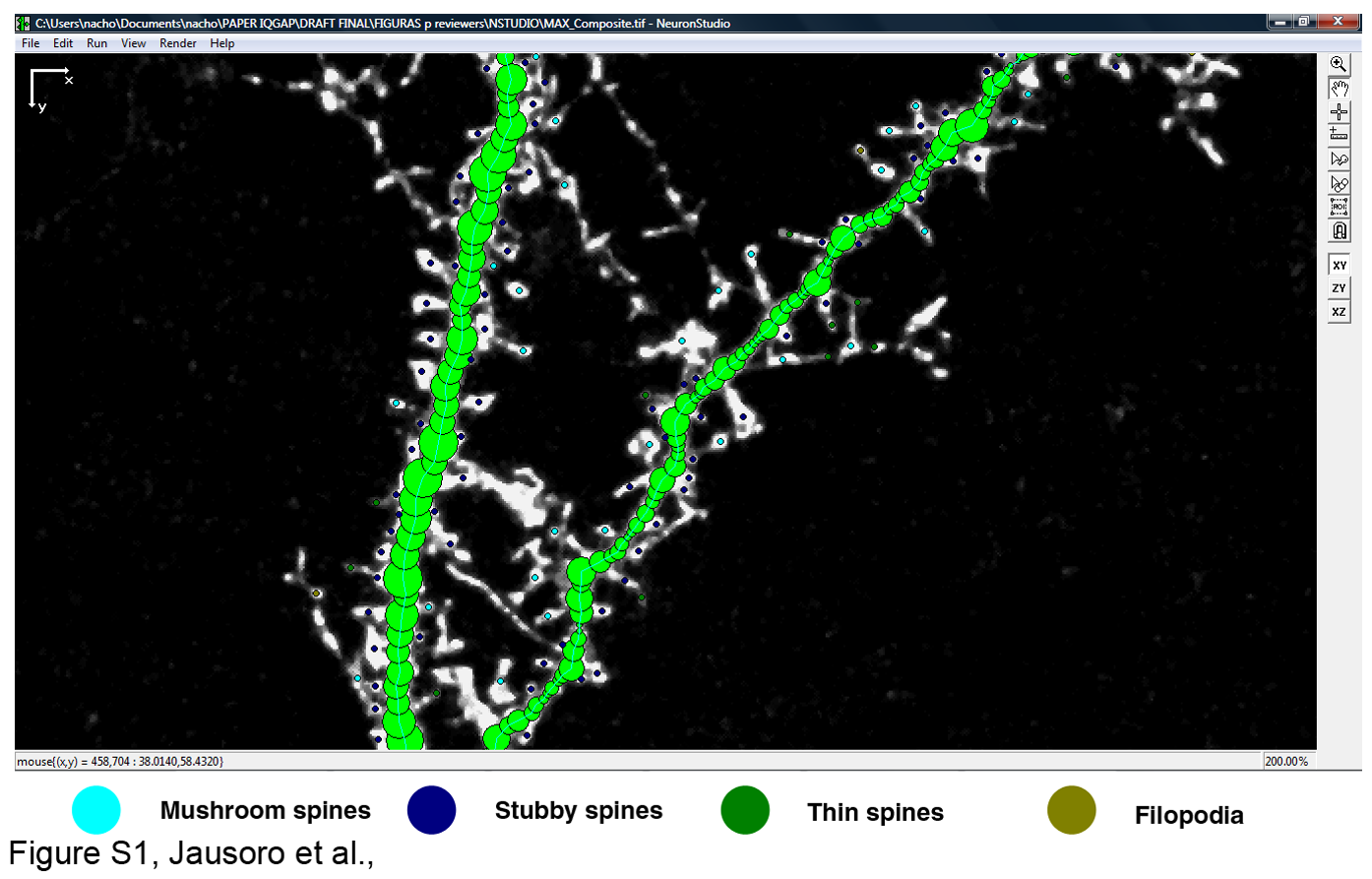

Supplement: Figure S1 — Computer-assisted analysis of spine number and shape. A representative image of the computer display provided by the software used to automatically evaluate changes in spine number and shape. For further details see Ref. 42 and 43. (TIFF) [file pone.0056574.s001.tif]

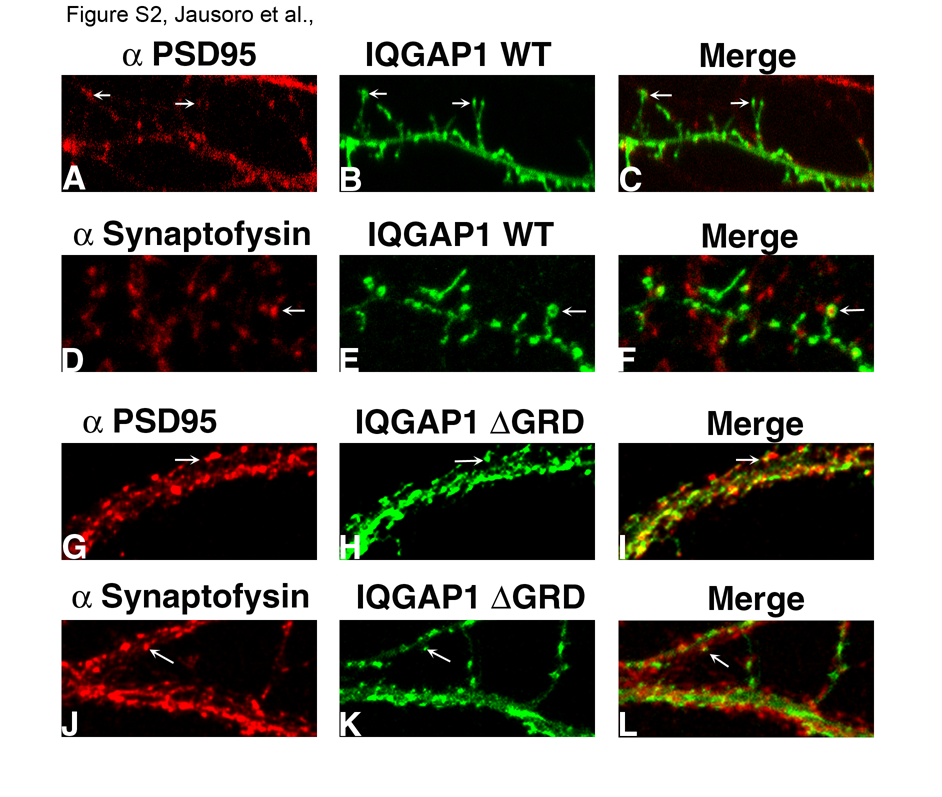

Supplement: Figure S2 — PSD95 immunofluorescence in neurons expressing IQGAP1 and its mutants. (A-C) High magnification views of a dendritic segment from a 17 DIV hippocampal cell culture transfected with myc-tagged IQGAP1 WT (green) and double stained with anti-PSD95 (red); note that long spines stain for PSD95 (arrows). (D-F). A similar set of images but from a culture transfected with myc-tagged IQGAP1 WT (green) and double stained with anti-synaptophysin (red); note that spines colocalize with endogenous synaptophysin puncta (arrows). (G-I) High magnification views of a dendritic segment from a 17 DIV cultured hippocampal neuron transfected with myc-tagged Δ-GRD IQGAP1 (green) and double stained with anti-PSD95 (red); note that stubby spines stain for PSD95 (arrows). (J-L) A similar set of images but from a 17 DIV cultured hippocampal neuron transfected with myc-tagged Δ-GRD IQGAP1 (green) and double stained with anti-synaptophysin (red); note that stubby spines colocalize with endogenous synaptophysin puncta (arrows). (TIFF) [file pone.0056574.s002.tif]

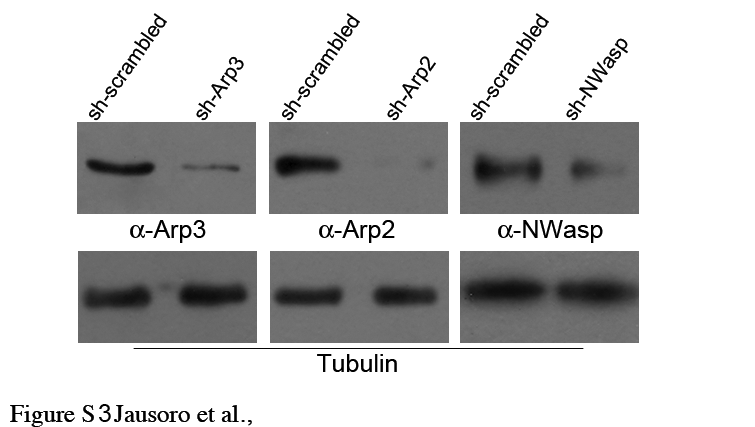

Supplement: Figure S3 — Short-hairpin RNAi suppression of Arp3, Arp2 and NWASP. Western blots showing levels of Arp3, Arp2, NWASP, and ß-tubulin in extracts of CHO cells treated with sh-Arp3, sh-Arp2, sh-NWASP or their corresponding scrambled oligonucleotides. For this experiment, cultures were transfected with the corresponding constructs and cell extracts obtained 24 hours later. (TIFF) [file pone.0056574.s003.tif]

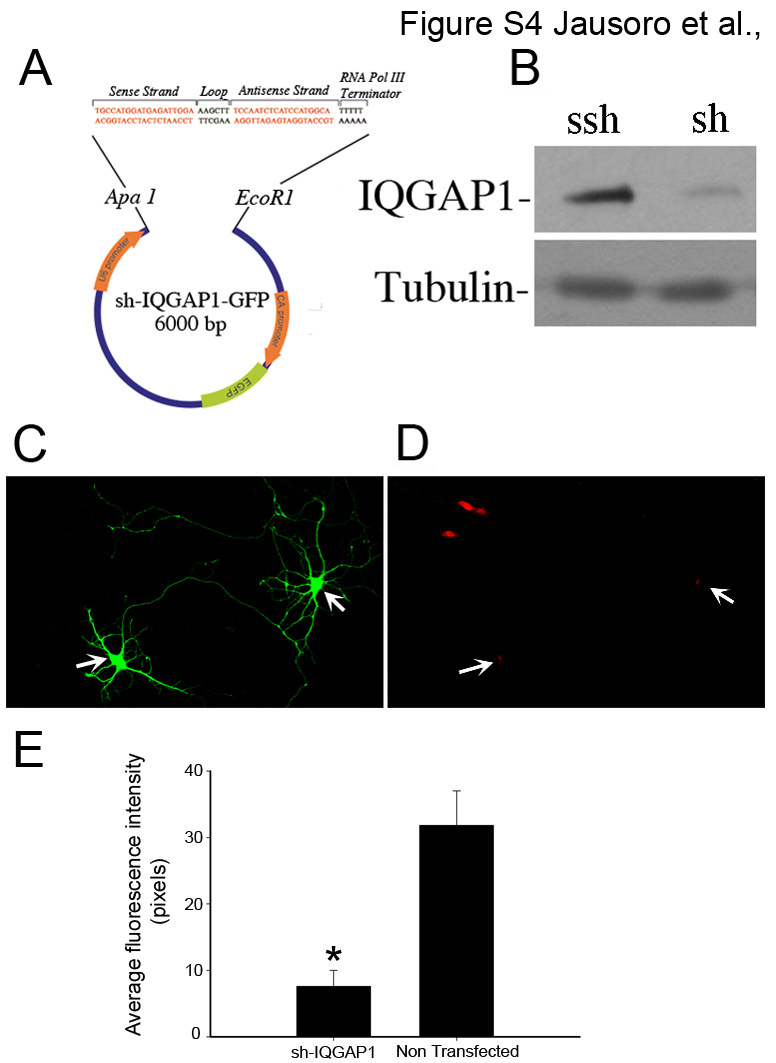

Supplement: Figure S4 — Short-hairpin RNAi suppression of IQGAP1. (A) Schematic representation of the sh-IQGAP1. (B) Western blots showing levels of IQGAP1 in extracts of CHO cells treated with sh-IQGAP1 or its corresponding scrambled oligonucleotide (ssh-IQGAP1). (C, D) Confocal images showing representatives images of cells expressing sh-IQGAP1-GFP (green) double stained with anti-IQGAP1. Note the decrease in IQGAP1 fluorescence in the cells expressing the RNAi (arrows). (E) Quantitative measurements of IQGAP1 fluorescence intensity in cultured hippocampal pyramidal neurons transfected with ssh-IQGAP1 or sh-IQGAP1. The values expressed in pixels represent the average fluorescent intensity within the cell body, initial, middle and distal neuritic segments. Note the significant reduction (P<0.001) in IQGAP1 fluorescent intensity in the RNAi-treated cultures. In all these experiments, fluorescent measurements were performed using 8-bites images. (TIFF) [file pone.0056574.s004.tif]

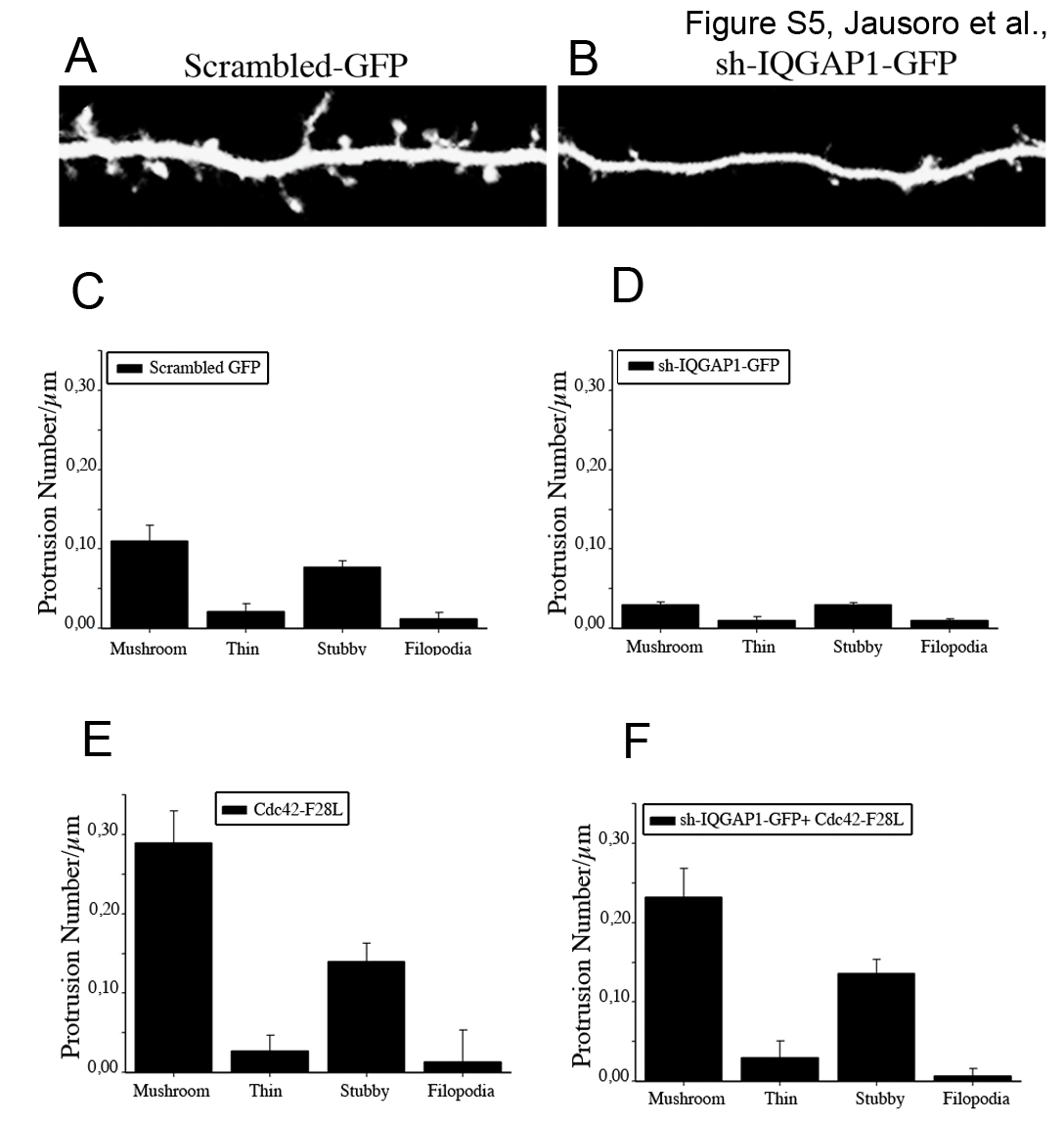

Supplement: Figure S5 — Cdc42 rescues the inhibitory effect of IQGAP1 suppression on spine number. (A) A dendritic segment of a neuron expressing ssh-IQGAP1-GFP (scrambled) oligonucleotide. (B) A dendritic segment of a neuron expressing sh-IQGAP1-GFP oligonucleotide; note the decrease in spine number. (C-F) Graphs showing the effect of IQGAP1 suppression on the number and type of dendritic protrusions. Note that a fast cycling Cdc42 (F28L) mutant rescues the decrease in spine number observed after IQGAP1 suppression. Bars represent mean ± standard deviation. (TIFF) [file pone.0056574.s005.tif]
