# Supplementary material for: Regulation of Spine Density and Morphology by IQGAP1 Protein Domains
Source: PLoS One. 2013 Feb 18;8(2):e56574. doi: 10.1371/journal.pone.0056574 (PMC3575492; doi:10.1371/journal.pone.0056574)
Supplement: Table S1 — A comparison of spine number and type as determined by manual counting vs. automated methods [42], [43] . Each value is the mean ± standard deviation. At least 10 dendritic segments (50 µm length/each) per cell (total 6 cells per culture) from at least 3 different cultures were evaluated. Note that both methods gave similar results. (DOC) [file pone.0056574.s006.doc]

**Table 1**. A comparion of spine number and type as determined by manual counting vs. automated methdos (Ref. 42, 43).

|  | Total Number/µm |
| --- | --- |
| Automated Detection | 0,24 ± 0,02/µm |
| Manual Detection | 0,22 ± 0,02/µm |

|  | Automated Detection | Manual Detection |
| --- | --- | --- |
| Mushroom | 0,13 ±0,01/µm | 0,12± 0,01/µm |
| Stubby | 0,9 ±0,01/µm | 0,8 ±0,03/ µm |
| Thin | 0,02/µm | 0,02/µm |
| Filopodia (other) | 0,01/µm | 0,01/µm |

| Head Diameter (for mushroon spines) | Spine Length (for mushroom spines) |
| --- | --- |
| 1.09± 0,2μm | 2.8 ± 0,2 μm |
| 1.18± 0,2 μm | 3.25 ± 0,4 μm |

Each value the mean ± \± standard deviation. At least 10 dendritic segments (50 µm length/each) per cell (total 6 cells per culture) from at least 3 different cultures were evaluated. Note that both methods gave similar results.
